# Administration of Dexmedetomidine inhibited NLRP3 inflammasome and microglial cell activities in hippocampus of traumatic brain injury rats

**DOI:** 10.1042/BSR20180892

**Published:** 2018-10-15

**Authors:** Bin Zheng, Shuncai Zhang, Yanlu Ying, Xinying Guo, Hengchang Li, Lixin Xu, Xiangcai Ruan

**Affiliations:** 1 Department of Anesthesiology, The First Affiliated Hospital of Jinan University, Guangzhou, China; 2 Department of Anesthesiology, Guangzhou First People’s Hospital, School of Medicine, South China University of Technology, Guangzhou, China; 3 Department of Anesthesiology, Jieyang People’s Hospital, Jieyang, China

**Keywords:** caspase-1, Dexmedetomidine, inflammation, NLRP3, traumatic brain injury

## Abstract

The abnormally high nucleotide-binding oligomerization domain (NOD)-like receptor family pyrin domain containing 3 (NLRP3) inflammasome activity is a typical characteristic of traumatic brain injury (TBI). Dexmedetomidine (Dex) is a highly selective α-2 adrenergic receptor agonist that inhibits the activation of NLRP3. Thus, it was hypothesized that Dex could attenuate TBI by inhibiting NLRP3 inflammasome activity in hippocampus. Rats were subjected to controlled cortical impact method to induce TBI, and treated with Dex. The effect of Dex treatment on the cognitive function, NLRP3 activity, and microglial activation in rat brain tissues was assessed. The administration of Dex improved performance of TBI rats in Morris water maze (MWM) test, which was associated with the increased neurone viability and suppressed microglia activity. Moreover, the administration of Dex inhibited the neuroinflammation in brain tissue as well as the expressions of NLRP3 and caspase-1. Additionally, Dex and NLRP3 inhibitor, BAY-11-7082 had a synergistic effect in inhibiting NLRP3/caspase-1 axis activity and improving TBI. The findings outlined in the current study indicated that the improvement effect of Dex on TBI was related to its effect on NLRP3 activity.

## Introduction

Traumatic brain injury (TBI) is a pathological event characterized by axonal destruction, neurone loss, and demyelination [1]. The disorder is the leading cause of mobility and morbidity in patients ageing from 1 to 44 [2,3] and has an annual incidence of 2.0–2.5 million in U.S.A. only. Although emergency management strategies for TBI are under development, patients surviving from the disorder still suffer from not only physical disability, but also neurobehavioral dysfunction [4], which all subsequently increase patients’ susceptibility to neurodegenerative diseases such as Alzheimer’s disease and Parkinson’s disease [5,6]. Currently, the etiologic mechanisms underlying the neuropsychiatric consequences of TBI are still partially resolved, but it is well recognized that neuroinflammation plays a crucial role in the pathogenesis of TBI and scientists and clinicians have regarded inflammation as a promising pharmacological target for treatment of TBI and its secondary injuries [7].

Neuroinflammation is a response initiated in innate immune system in central nervous system (CNS) and is important to tissue damages in CNS [8]. As being previously inferred, neuroinflammation can be both beneficial through supporting regenerative events and deleterious through amplifying local destructive pathways to neurone systems [9]. The latter effect of neuroinflammation is exerted by inducing local destructive pathways [9]. With the increasing understanding of inflammation, it is recently reported that the initiation of inflammatory response involves the participant of multiprotein complexes termed as ‘inflammasomes’ [10]. The assembly of an inflammasome can trigger proteolytic cleavage of dormant procaspase-1 into caspase-1, which then induces the production of mature and biologically active IL-1β and IL-18 [10]. Thus, inflammaosomes are well-characterized by their involvements in modulation of immune response to microbial infection and cellular damage [10]. Regarding its role in TBI, McKee and Lukens [11] have reported the up-regulation of inflammasome proteins after TBI. The formation of inflammasomes requires a pattern recognition receptor (PRR) as the sensor and to date, five PRR members, including nucleotide-binding oligomerization domain (NOD)-like receptor family pyrin domain containing 1 (NLRP1), NLRP3, NLRC4, Pyrin, and AIM2 have been shown to participate in the formation of inflammasomes [12,13]. Of different inflammasome types, NLRP3 inflammasome has drawn most attention in that the involvement of NLRP3 is highlighted in the development in Alzheimer’s disease [14], Huntington’s disease [15], pneumococcal meningitis [16], and TBI [17]. Thus, the inhibition of NLRP3 inflammasome formation might be a potential treatment strategy for TBI.

In previous studies, several agents have been employed for treatment of brain injury by inhibiting inflammasome activity. For example, Fan et al. [7] show that mangiferin attenuates TBI by inhibiting NLRP3 inflammasome. In the study by Ismael et al. [18], it is reported that selective NLRP3 inflammasome inhibitor can protect mice against TBI. Based on these reports, the application of inflammasome inhibitor has shown their value in the treatment of TBI. Dexmedetomidine (Dex) is a highly selective α-2 adrenergic receptor agonist [19]. The drug has been utilized in the patients with TBI and shows considerable treatment effect. However, whether its anti-TBI effect is exerted by inhibiting NLRP3 inflammasome activity is not fully explored. Combined the above information together, the current study hypothesized that Dex rehabilitated cognitive function in TBI patients by inhibiting NLRP3 inflammasome activity in hippocampus.

Therefore, to verify the hypothesis, rats were subjected to TBI induction and then treated with Dex. The cognitive function, tissue structure, and neurone viability in hippocampus of rat models were then assessed. Moreover, the treatment of Dex on NLRP3 inflammasome activity was explored as well. Collectively, the findings outlined in the current study showed that the cognitive improvement effect of Dex on TBI was related to its inhibition of NLRP3 inflammasome activity in hippocampus.

## Materials and methods

### Controlled cortical impact model and animal group

Male Sprague–Dawley (SD) rats were provided by Medical Laboratory Animal Center of Guangdong Province. The present study was approved by the Ethics Committee of The First Affiliated Hospital of Jinan University and handled in accordance with the guidelines on animal care of the First Affiliated Hospital of Jinan University. The rats were housed in a 12:12-h light–dark cycle at a 22°C and had free access to food and water. TBI symptoms were induced in mice using controlled cortical impact model: briefly, rats were anesthetized with 1% pentobarbital sodium (50 mg/kg) and fixed on a stereotaxic in a prone position. Then, an incision was made on the skin of cranial vault and a 6-mm hole was made on the line connecting bregma and lambdoidal suture. Cortical contusion at parietal lobe of left cerebral cortex was produced by a 3.5 mm/s-2 mm impact. Afterward, injury was stanched and sutured. The establishment of TBI model was assessed using neurological deficit score (NIHSS) according to the study of Vakili et al. [20]. Compared with control healthy rats (NIHSS = 0), rats underwent controlled cortical impact administration (NIHSS = 3.0 ± 0.25) showed a significant increase in NIHSS, indicating the successful induction of TBI symptoms.

#### Protocol I

Eighty rats were randomly divided into four groups (20 rats for each group) for demonstrating the effect of Dex on TBI progression. Control group, healthy male SD rats were intraperitoneally injected with 1 ml of 0.9% normal saline (NS) for four consecutive days. TBI group, SD rats were first subjected to controlled cortical impact model induction and then intraperitoneally injected with 1 ml of 0.9% NS for four consecutive days after the surgery. DEX group, healthy male SD rats were intraperitoneally injected with 1 ml of 20 μg/kg Dex [21] (12120125, Jiang Su Heng Rui Medicine Co., Ltd, China) for four consecutive days. DEX+TBI group, SD rats were first subjected to controlled cortical impact model induction and then intraperitoneally injected with 20 μg/kg Dex for four consecutive days after the surgery.

#### Protocol II

Thirty rats were randomly divided into five groups to explain the role of NLRP3 in the brain protection effect of Dex (six for each group). Control group, healthy male SD rats were intraperitoneally injected with 1 ml of 0.9% NS for four consecutive days. TBI group, SD rats were first subjected to controlled cortical impact model induction and then intraperitoneally injected with 1 ml of 0.9% NS for four consecutive days after the surgery. DEX+TBI group, SD rats were first subjected to controlled cortical impact model induction and then intraperitoneally injected with 20 μg/kg Dex for four consecutive days after the surgery. TBI+BAY-11-7082 group, SD rats were first subjected to controlled cortical impact model induction and then intraperitoneally injected with NLRP3 inhibitor BAY-11-7082 (20 mg/kg) [22]. TBI+DEX+BAY-11-7082 group, SD rats were first subjected to controlled cortical impact model induction and then intraperitoneally injected with 20 μg/kg of DEX+BAY-11-7082.

### Morris water maze test

For ten randomly selected rats in Protocol I, 7 days after the model induction. Morris water maze (MWM) test was used to assess the effect of Dex administration on the cognitive function of rats. The assays were performed routinely as reported previously [23,24] by two investigators blinded to the experiment designs. In brief, for visible platform trail in 60 s, rats were allowed to swim for 60 s before getting to the platform for 5 days. For probe trial in 60 s, the time through the quadrant of the former platform position was measured. The track images were captured using Anymaze Software (ANY-maze, U.S.A.).

### ELISA

Rats were killed with overdose pentobarbital sodium after the 7-day house and hippocampus tissues were collected. The production of IL-1β (E-EL-R0012c, Elabscience Biotechnology, Wuhan, China) and IL-6 (E-EL-R0015c, Elabscience Biotechnology, Wuhan, China) in hippocampus tissues was detected using corresponding ELISA kits according to the manufacturer’s instructions: briefly, hippocampus tissues were homogenized and then 100-μl sample was incubated in one well of ELISA plate at 37°C for 90 min Afterward, 100 μl biotinylation antibody was added into the well and incubated at 37°C for 60 min. Then 100 μl enzyme-binding solution was added into the mixture and incubated at 37°C for 30 min. Finally, 50 μl TMB substrate was added and incubated at 37°C for 30 min. The OD values at 450 nm of different samples were detected using a Microplate Reader (MULTISKAN MK3, Thermo).

### Immunofluorescent assay

Hippocampus tissues were fixed with 4% paraformaldehyde and then washed with PBS for three times. Afterward, sections were incubated with primary antibodies against NeuN (1:500, ab177487, Abcam, U.S.A.), Iba-l (1:500, ab15690, Abcam, U.S.A.), NLRP3 (1:600, ab214185, Abcam, U.S.A.), and caspase-1 (1:500, ab62698, Abcam, U.S.A.) at 4°C overnight. Upon completion of the culture, secondary Cy3-labeled IgG antibodies (1:200, red, A21216, Jackson ImmunoReaserch, U.S.A.) and FITC-labeled IgG antibodies (1:1000, green, ab6785, Abcam, U.S.A.) were added into the cell cultures and incubated for 1 h in dark. Then sections were stained with DAPI and washed using PBS for three times. The images were captured with a fluorescent microscopy (Leica, Germany) at 400× magnification.

### Nissl staining

Viability of neurones in hippocampus tissues was detected with Nissl staining. Sections were stained with Methylene Blue buffer for 10 min and then put into acetic acid buffer for 2 min. The plasma of stained neurone was blue. The images were captured under a microscope at 400× magnification.

### Western blotting

Hippocampus tissues were lysed using RIPA protein lysis buffer and total protein was collected by centrifugation. Protein concentration was determined using BCA method according to the manufacturer’s instructions. Protein samples (2 μg/μl) were subjected to SDS/PAGE and then transferred on to PVDF membranes. After transfer, membranes were incubated with the primary antibodies against NLRP3 (1:2000, ab51952, Abcam, U.K.), caspase-1 (1:1000, sc1218, Santa Cruz Biotechnology, U.S.A.), and β-actin (1:10000, 3700, Cell Signaling Technology, U.S.A.) at 4°C overnight and then incubated with secondary horseradish peroxidase-conjugated IgG antibodies (1:5000) at 37°C for 2 h. The protein bands were developed using the Beyo electrochemiluminescence Plus reagent and the images were captured in the Gel Imaging System (WD-9413B, Liuyi Factory, China).

### Statistical analysis

Data were expressed as mean ± S.D. One-way ANOVA and *post hoc* multiple comparisons using least square difference method were performed using GraphPad Prism version 6.0 (GraphPad Software, Inc., San Diego, CA) with a significant level of 0.05 (two-tailed *P*-value).

## Results

### Administration of Dex improved cognitive function of TBI rats

**Figure 1:**
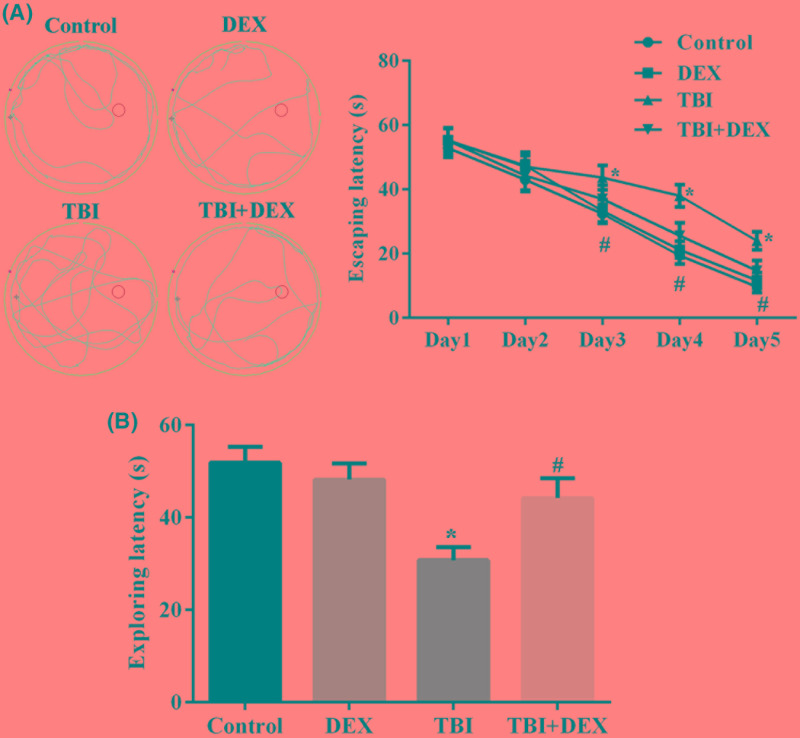
Administration of Dex improved cognitive function of TBI rats (**A**) Representative images of tracks and quantitative analysis result of rat escaping latency in MWM tests. (**B**) Quantitative analysis result of exploring latency in MWM tests. *P<0.05 compared with Control group. ^#^*P*<0.05 compared with TBI group.

TBI symptoms were induced in SD rats using controlled cortical impact method. Seven days after the model inductions, ten randomly selected rats in each group were subjected to MWM experiment to assess the influence of model induction as well as Dex administration on cognitive function of the rats. As shown in Figure 1A, no significant difference was observed amongst groups during the first two trials. However, since the third day, rats with TBI showed a significantly higher escaping time compared with the other three groups, including those with TBI symptoms but treated with Dex (*P*<0.05) (Figure 1A). Moreover, it was also found that Dex administration had no influence on the cognitive function of healthy rats, confirming the safety of the drug. Similar to the changes in escaping time, TBI surgery also significantly decreased the exploring time of rats in former platforms and the administration of Dex increased the time in TBI rats (Figure 1B), which further supported the improving effect of Dex on cognitive function impaired by TBI.

### Administration of Dex increased neurone viability, inhibited microglia activation, and suppressed inflammation in hippocampus tissues

**Figure 2:**
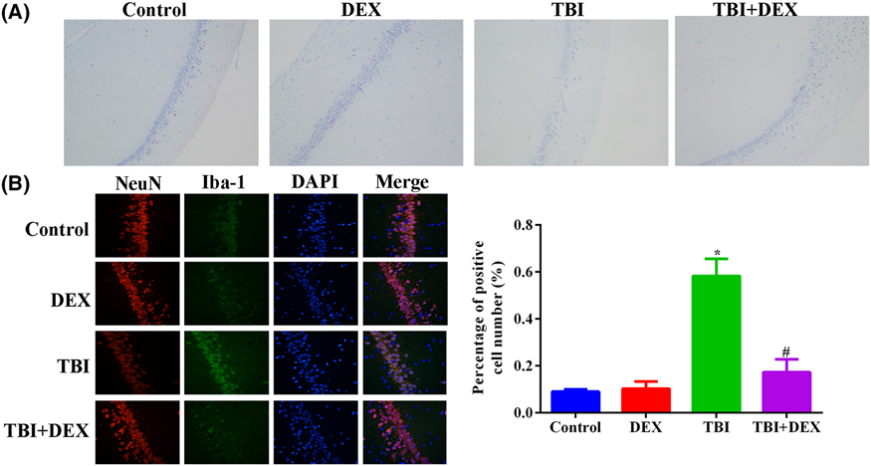
Administration of Dex increased neurone viability and suppressed microglia activation in hippocampus tissues (**A**) Representative images of Nissl staining of neurones in hippocampus tissues. (**B**) Representative images and quantitative analysis result of immunofluorescence detection of Iba-1 in hippocampus tissues. *P<0.05 compared with Control group. ^#^*P*<0.05 compared with TBI group. Magnification: 400×.

**Figure 3:**
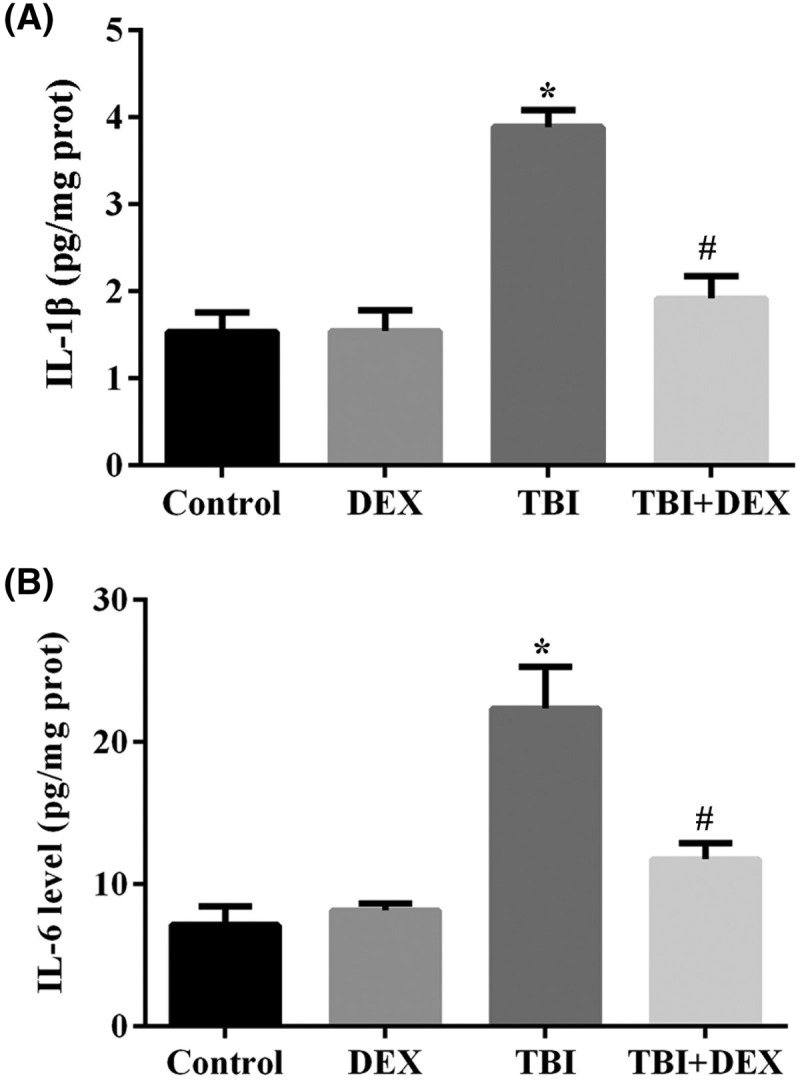
Administration of Dex inhibited pro-inflammation cytokine production in hippocampus tissues (**A**) Quantitative analysis result of ELISA detection of IL-1β production in hippocampus tissues. (**B**) Quantitative analysis result of ELISA detection of IL-6 production in hippocampus tissues. *P<0.05 compared with Control group. ^#^*P*<0.05 compared with TBI group.

The number of neurones in hippocampus tissues was measured using Nissl staining. The induction of TBI model significantly decreased the average neurone number in rats, which could be restored by Dex administration (Figure 2A). Compared with neurones, the TBI surgery induced microglia activation in hippocampus, which was represented by the higher number of Iba-l positive cells (Figure 2B), but in TBI rats treated with Dex, the activation of microglia was dramatically suppressed. Associated with the changes in neurone viability and microglia activity, the production of IL-1β and IL-6 in hippocampus tissues was first induced by TBI surgery and then suppressed by Dex administration (Figure 3A,B), evidently indicating that inflammation associated with TBI was inhibited by Dex treatment.

### Administration of Dex decreased inflammasome activity in hippocampus tissues

**Figure 4:**
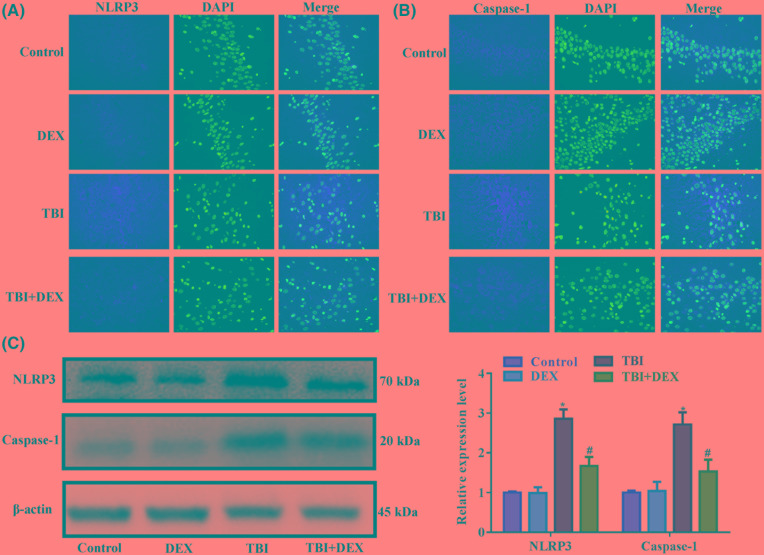
Administration of Dex inhibited NLRP3 and caspase-1 expressions in hippocampus tissues (**A**) Representative images of immunofluorescence detection of NLRP3 in hippocampus tissues. (**B**) Representative images of immunofluorescence detection of caspase-1 in hippocampus tissues. (**C**) Representative images and quantitative analysis result of Western blotting detection of NLRP3 and caspase-1 in hippocampus tissues. *P<0.05 compared with Control group. ^#^*P*<0.05 compared with TBI group. Magnification: 400×.

Initiation of inflammatory response involves the participant of inflammasomes. In the current study, we focussed on the activity of NLRP3-mediated inflammasome to explore the mechanism driving the anti-TBI effect of Dex. The immunofluorescence (Figure 4A,B) and Western blotting detections (Figure 4C) showed that the induction of TBI increased the expressions of NLRP3 and caspase-1 in hippocampus tissues, indicating that the activity of inflammasomes was increased by brain injuries. However, in TBI rats treated with Dex, the expressions and distributions of NLRP3 and caspase-1 were both restricted (Figure 4A–C), implying that the anti-TBI effect of Dex might be related to its inhibition on NLRP3-mediated inflammasomes.

### Dex exerted its cognitive improvement function by inhibiting NLRP3-mediated inflammasomes activity

**Figure 5:**
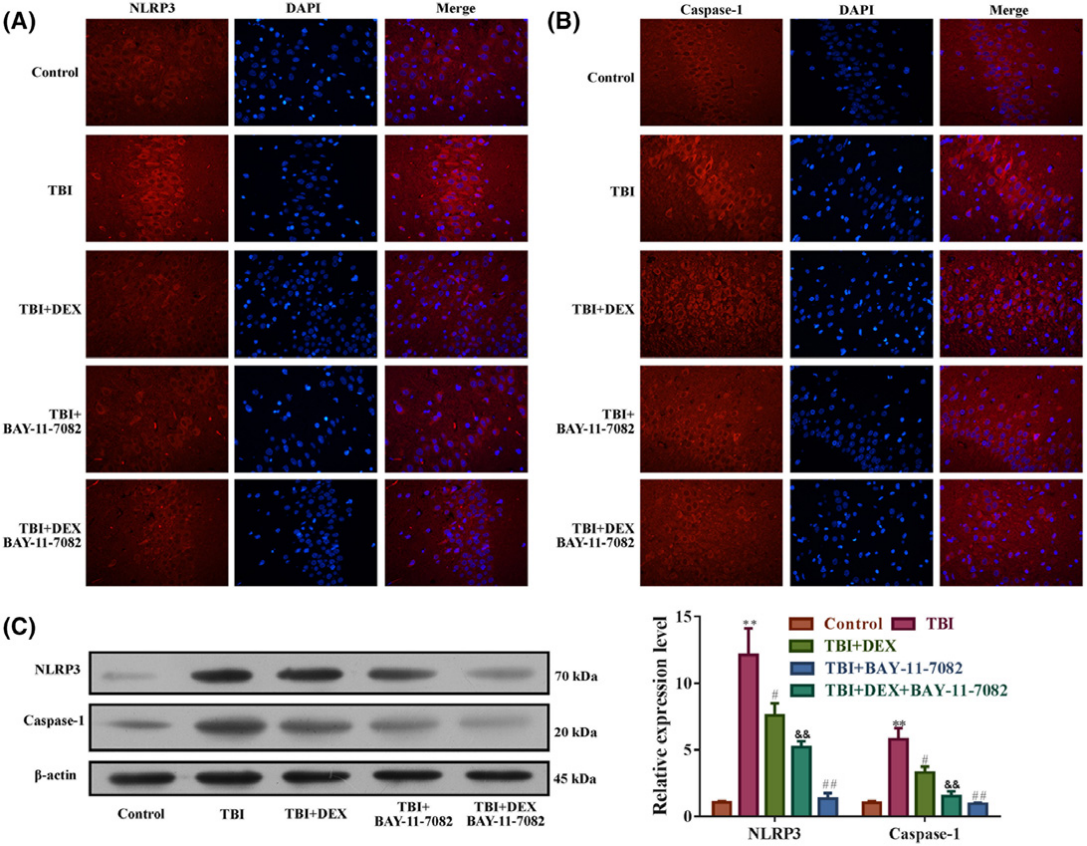
Administration of Dex and BAY-11-7082 had a synergistic effect in inhibiting NLRP3/caspase-1 pathway (**A**) Representative images of immunofluorescence detection of NLRP3 in hippocampus tissues. (**B**) Representative images of immunofluorescence detection of caspase-1 in hippocampus tissues. (**C**) Representative images and quantitative analysis results of Western blotting detection of NLRP3 and caspase-1 in hippocampus tissues. **P<0.01 compared with Control group. ^#^*P*<0.05 compared with TBI group. ^##^*P*<0.01 compared with TBI group. ^&&^*P*<0.01 compared with TBI+DEX group. Magnification: 400×.

**Figure 6:**
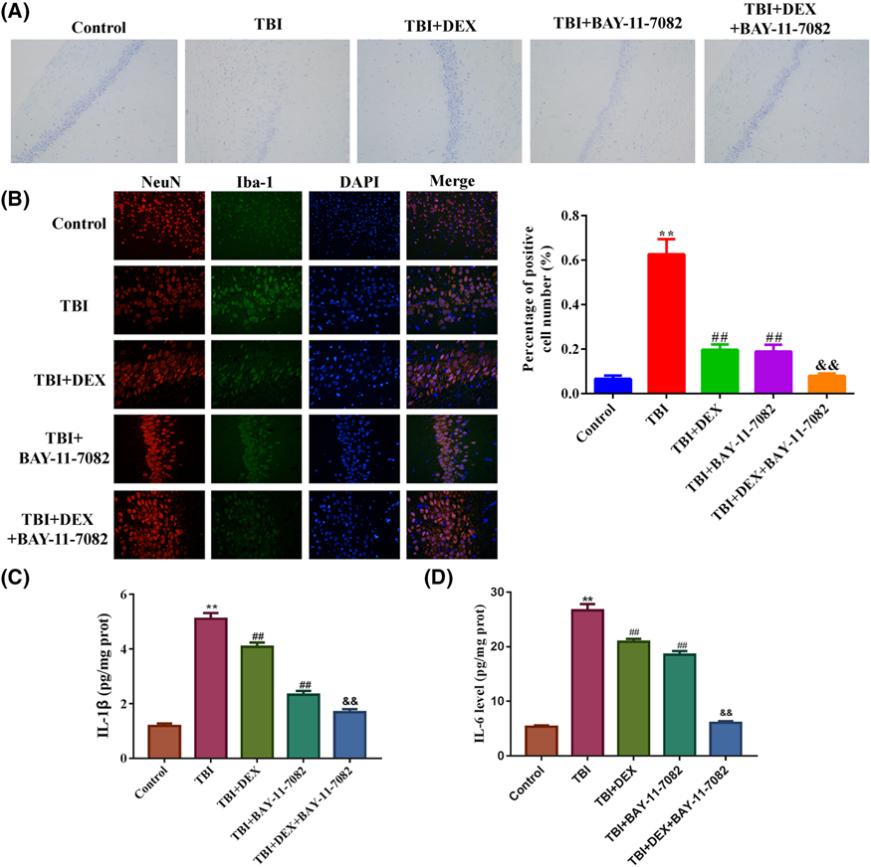
Administration of Dex and BAY-11-7082 had a synergistic effect in increasing neurone viability and suppressing microglia activation as well as inflammation in hippocampus tissues (**A**) Representative images of Nissl staining of neurones in hippocampus tissues. (**B**) Representative images and quantitative analysis result of immunofluorescence detection of Iba-1 in hippocampus tissues. (**C**) Quantitative analysis result of ELISA detection of IL-1β production in hippocampus tissues. (**D**) Quantitative analysis result of ELISA detection of IL-6 production in hippocampus tissues. **P<0.01 compared with Control group. ^##^*P*<0.01 compared with TBI group. ^&&^*P*<0.01 compared with TBI+DEX group.

To confirm the inhibitory effect of Dex on NLRP3-mediated inflammasome activity, rats in TBI and TBI+Dex groups were further injected with NLRP3 inhibitor BAY-11-7082. The administration of BAY-11-7082 significantly inhibited the expressions of NLRP3 and caspase-1 induced by TBI surgery in hippocampus tissues (Figure 5A–C), which was associated with increased neurone number (Figure 6A), and suppressed microglia activation (Figure 6B) and inflammation (Figure 6C,D). The effect was comparable with that of Dex administration. Moreover, the co-administration of Dex and BAY-11-7082 had a synergistic effect on the expressions of NLRP3 and capase-1 as well as on the neurone viability (Figure 6A), microglia activation (Figure 6B), and inflammation (Figure 6C,D).

## Discussion

As a specific agonist of α2-adrenoceptor, Dex has shown its pharmacological functions including sedation, analgesia, and anxiolysis [25], and has been widely employed for calmness in tracheal intubation and mechanical ventilation [26]. More recently, several reports also demonstrated the potential of Dex in protecting multiple organs against damages caused by inflammation, oxidation, and apoptosis [27–29]. Therefore, emerging studies have paid attention to the potential of Dex beyond its anesthetic function. The aim of the current study was to explore the possible mechanism driving the brain protection effect of Dex in TBI. In fact, the brain protection effect of Dex has been proved by several studies. For example, in the study of Schoeler et al. [30], it is reported that Dex protects organotypic hippocampal slice cultures against focal mechanical trauma by activating extracellular signal-regulated kinase (ERK) signaling. Similarly, Wu et al. [31] also report that Dex improves mice motor function by reducing tissue loss and cell death caused by TBI. In the current study, TBI symptoms were induced in mice using controlled cortical impact method. Then, the TBI mice were treated with Dex. The results of MWM experiments and histological stains showed that the administration of Dex improved the cognitive function and brain structure of TBI mice. The improved behavior in TBI mice was associated with the maintained neurone viability and suppressed microglia activation in hippocampus tissues. These results confirmed the neuroprotection effect of Dex, which was in consistence with previous studies [31–33].

Dex can exert its neuroprotection effect via multiple molecular mechanisms. As mentioned above, the agent can activate ERK pathways, which may contribute to the reduction in cell apoptosis in neurones [30]. ERK is a key enzyme in cell metabolism activated by many different types of tissue injury and has been attributed a ‘survival’ function. The activation of survival signals itself can cause cells to exist from apoptosis. In addition, Dex can also inhibit neuronal apoptosis by up-regulating heat shock protein 70 (HSP70) expression in hippocampus [33]. HSP70 is a highly conserved molecular chaperone and confers cellular protection against insults by possessing potent anti-apoptotic properties [34]. Except for the anti-apoptosis effect, Dex is also a promising anti-inflammation agent and several Dex-based treatments are developed based on this potential. In previous studies, Dex treatments are proved to attenuate hyperoxia-induced acute lung injury and liver injury by inhibiting the activation of NLRP3 inflammasomes [35,36]. It is well recognized that the activation of NLRP3 inflammasomes is an indicator for the development of neurodegeneration diseases [14–17] and also a pre-requisite for neuroinflammation initiation [10]. Thus, we hypothesized that the anti-TBI function of Dex might be related to the inhibition of NLRP3 inflammasome activation.

To verify this hypothesis, we examined the inflammatory response in model mice. Along with the improvement in cognitive function and hippocampus tissue structure, the production of IL-1β and IL-6 was suppressed by Dex. Moreover, the expressions of NLRP3 and caspase-1 were inhibited by Dex as well. The NLRP3 inflammasomes can be activated by ATP, bacterial toxins, endogenous molecules, and particulate matters [37–40]. Subsequently, the assembly of inflammasomes induce the proteolytic cleavage of dormant procaspase-1 into active caspase-1 and promotes the production of pro-inflammation cytokines [41,42]. The inhibited production of cytokines and expressions of NLRP3 and caspase-1 reported by our study inferred the possibility that Dex protected mice against TBI by inhibiting NLRP3-mediated inflammasome activation, which then suppressed the expression of caspase-1. Based on the subsequent assays using NLRP3 inhibitor BAY-11-7082, it was found that the inhibiting effect of Dex on NLRP3/caspase-1 axis was comparable with that of NLRP3 inhibitor and the co-administration of both agents could further block NLRP3/caspase-1 signaling transduction. Moreover, the synergistic effect of the two agents was also observed in the detections of neurone viability, microglia activation, and cytokine production, which indicated that the anti-TBI function of Dex was dependent on the inhibition of NLRP3/caspase-1 pathway in hippocampus tissues.

Collectively, the current study confirmed the protection effect of Dex against TBI. The effect might be related to its inhibition on the formation of NLRP3-mediated inflammasome. However, our study only showed that the inhibiting effect of Dex on NLRP3/caspase-1 axis was comparable with that of BAY-11-7082 and the two agents had a synergistic blocking effect on NLRP3/caspase-1 signaling, further exploration on the mechanism driving the anti-inflammation effect of Dex in the treatment of TBI are needed.

## Abbreviations

CNS: central nervous system
Dex: dexmedetomidine
ERK: extracellular signal-regulated kinase
HSP70: heat shock protein 70
Iba: ionized calcium-binding adapter molecule
IL: interleukin
MWM: Morris water maze
OD: optical density
NIHSS: The National Institute of Health Stroke Scale
NeuN: neuronal nuclei
NLRP3: nucleotide-binding oligomerization domain-like receptor family pyrin domain containing 3
NS: normal saline
PRR: pattern recognition receptor
RIPA: radioimmunoprecipitation assay
SD: Sprague–Dawley
TBI: traumatic brain injury
TMB: tetramethylbenzidine
